# Gastroprotective and ulcer healing effects of *piptadeniastrum Africanum* on experimentally induced gastric ulcers in rats

**DOI:** 10.1186/s12906-015-0713-5

**Published:** 2015-07-08

**Authors:** Gilbert Ateufack, Elisabeth Carol Domgnim Mokam, Marius Mbiantcha, Rostand Breuil Dongmo Feudjio, Nana David, Albert Kamanyi

**Affiliations:** Laboratory of Animal Physiology and Phytopharmacology, Faculty of Science, University of Dschang, P.O. Box 67, Dschang, Cameroon; Laboratory of Animal Biology and Physiology, Faculty of Science, University of Yaoundé 1, P.O. Box 812, Yaoundé, Cameroon; Department of Animal Biology, Dschang University, Animal Physiology and Phytopharmacology Laboratory, P.O. Box 67, Dschang, Cameroon

**Keywords:** Gastric ulcers, Gastroprotective, *Piptadeniastrum africanum*

## Abstract

**Background:**

Gastric peptic ulcer is one of the common disorders of gastrointestinal tract, which occur due to an imbalance between the offensive and defensive factors. It is an illness that affects a considerable number of people worldwide. This study was conducted to evaluate the antiulcerogenic and antiulcer effects and recognize the basic mechanism of action of *Piptadeniastrum africanum* stem bark extracts.

**Methods:**

The aqueous and methanol extracts of *Piptadeniastrum africanum* were administered at the doses 125, 250 and 500 mg/kg to evaluate their effects on gastric ulcer induced by the HCl/ethanol mixture, indomethacin and acetic acid in Wistar strain male adult rats, aged between 12 and 16 weeks and weighing between 180 and 220 g. Ranitidine, Maalox and Misoprostol were used as standard drugs. Histopathological examination and nitric oxide level were performed to evaluate the basic mechanism of action of *Piptadeniastrum africanum*. Phytochemical screening was carried out to identify known phytochemicals present in these extracts.

**Results:**

The aqueous and methanol extracts of stem bark of *Piptadeniastrum africanum* significantly inhibited (*p* < 0.01) gastric ulceration induced by HCl/ethanol to the percentages of inhibition of 81.38; 98.75 and 100 % for the aqueous extract and then 75.83, 89.76 and 96.52 % for the methanol extract, and with the Indomethacin-induced ulcers, aqueous and methanol extracts of bark of *Piptadeniastrum africanum* reduce significantly (*p* < 0.01) induced gastric lesions in rats, with percentage of cure 35.75; 52.33 and 98.58 % for the aqueous extract, and 33.7; 51.97; and 65.93 to the methanol extract. The results revealed a significant reduction of ulcerated surface in both extracts and increase of nitric oxide (NO) level with methanol extract. When compared to methanol extract, aqueous extract showed more pronounced effects, corresponding to percentages of healing of 59. 92; 84.12 and 59.65 % for the aqueous extract; and 70.43; 55.49 and 57.59 % for the methanol extract in the ulcer induced by acetic acid, all at the respective doses of 125, 250 and 500 mg/kg. Histopathological observations also demonstrated curative effect. As such, both extracts were found to exhibit preventive and curative effects through the release of NO and growth factors. This could also be due to the presence of phytochemicals such as alkaloids, flavonoids, phenols and saponins which act as antisecretory agents.

**Conclusions:**

*Piptadeniastrum africanum* stem bark extracts thus have gastroprotective and ulcer healing effects, which could result from their activities by stimulating important cellular mechanisms such as migration and proliferation of epithelial cells that may have a cytoprotective effect by stimulating the release of prostaglandins. These results are required to confirm the ethnopharmacological use of *Piptadeniastrum africanum* stem bark in the treatment of ulcer.

## Background

Gastric peptic ulcer is one of the common disorders of gastrointestinal tract, which occur due to an imbalance between the offensive (gastric acid secretion) and defensive (gastric mucosal integrity) factors [1]. It is an illness that affects a considerable number of people worldwide. The etiological factors of this disorder include: stress, smoking, nutritional deficiencies, infections, frequent and indiscriminate use of non-steroidal anti-inflammatory drugs (NSAIDs) [2].

A number of drugs including proton pump inhibitors and H2 receptor antagonists are available for the treatment of gastric ulcer, but clinical evaluation of these drugs have shown incidence of relapses, side effects and drug interactions [3]. Thus, there is an urgent need to identify more effective and safe antiulcer agent. In this context, the use of medicinal plants for the prevention and treatment of different pathologies is in continuous expansion worldwide [2]. In order to enhance the use of plants as potential sources of new therapeutic agents and to advocate for effective treatment of diseases with fewer side effects, it is necessary to carry out pharmacological and toxicological studies on plants used by traditional healers. To contribute to this program of study, a plant used in the treatment of several diseases in the Central Region of Cameroon caught our attention.

This is *Piptadeniastrum africanum* belonging to the family Mimosaceae and whose stem bark decoction is commonly used in traditional medicine in the treatment of inflammation and gastric ulcer [4]. Previous studies have reported that the plant possess analgesic and anti-inflammatory properties of bark [5] and the phytochemicals methanol extract of stem bark showed the presence of flavonoids and alkaloids [6] which have a significant effect in the treatment of gastric ulcer. However, there is no data reported on antiulcer and antiulcerogenic activities within the plant. Hence, the current study was undertaken to evaluate the antiulcerogenic and anti-ulcer properties of aqueous and methanol extracts of stem bark *Piptadeniastrum africanum*. Antiulcerogenic activity was tested through HCl-ethanol-induced model and antiulcer activity was evaluated through indomethacin-induced and acetic acid-induced models in rats. Histological assessment and NO levels were associated to evaluate the possible mechanism and healing process of the plant.

## Methods

### Collection and preparation of plant material

The plant that was used in this study is *Piptadeniastrum africanum* (Hook. f.) Family Mimosaceae harvested in the Central Region of Cameroon, district Bokito and authenticated at the National Herbarium in Yaounde (Cameroon) through a comparison with the voucher specimen No. 12115/SRF. A voucher specimen has been deposited at the Botany Department, University of Dschang. The collected fresh stem bark was scrapped, chopped, shade dried and coarsely powdered.

### Preparation of aqueous extract

Powdered *Piptadeniastrum africanum* stem bark (560 g) was boiled in 3 l of distilled water for 20 min. The decoction was taken and allowed to cool for 30 min at room temperature (24 ± 2 °C). The decoction was filtered through a Whatman filter paper no.1 and evaporated to dryness in an air oven at 40 °C to give 19.72 g of aqueous extract corresponding with an extraction yield of 3.25 %.

### Extraction of the methanol plant material

200 g of the stem bark powder was soaked with 1.5 l of methanol for 72 h. The filtrate was concentrated to dryness in a rotary evaporator under reduced pressure at a temperature of 65 °C to give 18.81 g of methanol extract (28.08 % yield).

### Preliminary phytochemical screening of extracts

Qualitative chemical tests were conducted for aqueous and methanol extracts to identify the various phytoconstituents. The aqueous and methanol extracts gave positive test for saponins, tannins, phenolic compounds, terpenoids and flavonoids [6].

### Chemicals and drugs

HCl, ethanol, acetic acid and Griess reagent were obtained from laboratory of animal physiology and phytopharmacology of the University of Dschang. Maalox® (Aluminium hydroxide plus magnesium hydroxide), Ranitidine® and Misoprostol® were purchased from a pharmacy. All other used chemicals and reagents were of analytical grade.

### Animals

The experiments were carried out on Wistar strain male adult rats, aged between 12 and 16 weeks and weighing between 180 and 220 g. The rats were raised in the animal house of the Faculty of Science of the University of Dschang and fed with normal laboratory rat diet; with feed and water given *ad libitum*. Prior to experimental protocol, the rats were acclimatized for 48 h to laboratory conditions for minimizing any nonspecific stress.

All procedures described in the present work as concerns the use of experimental animals were in strict respect of the ethics regarding the use, the handling and preservation of the Cameroonian flora and fauna as specified by the Ethics Committee of the Cameroon Ministry of Scientific Research and Technology, which has adopted the guidelines established by the European union on animal care and experimentation (CCE Council 86/609).

### Anti-ulcer trials

#### HCl/ethanol-induced gastric mucosal lesions

Gastric mucosal lesions were induced in male rats using the HCl/ethanol method as described by [7]. The animals were starved for 48 h and divided into nine groups consisting of six rats per group. Group 1 represented the negative control group, which received 1 mL/100 g body weight distilled water. Groups 2 and 3 represent the positive control groups which received 1 mL/100 g body weight Maalox (50mg/kg) and Ranitidine (50mg/kg) solutions, respectively. Groups 4 to 6 received 1mL/100 g body weight aqueous extract at doses of 125, 250 and 500mg/kg respectively. Groups 7 to 9 received 1 mL/100 g body weight methanol extract at doses of 125, 250 and 500mg/kg. All the test substances were administered orally. One hour after drug treatment, 1mL per 150 g body weight of the necrotizing solution (150mM HCl in 60 % ethanol) was given *per os* to each rat. The rats were sacrificed one hour later and the stomach removed and observed for ulcers in the glandular region. The surface area of each lesion was measured and scored as described by [8]. The ulcer index for each rat was taken as the mean ulcer score (0: no ulcer; 1: US ≤ 0.5mm^2^; 2: 0.5mm^2^ < US ≤ 2.5mm^2^; 3: 2.5mm^2^ < US ≤ 5mm^2^; 4: 5mm^2^ < US ≤ 10mm^2^; 5: 10mm^2^ < US ≤ 15mm^2^; 6: 15mm^2^ < US ≤ 20mm^2^; 7: 20mm^2^ < US ≤ 25mm^2^; 8: 25mm^2^ < US ≤ 30mm^2^; 9: 30mm^2^ < US ≤ 35mm^2^; 10:US > 35mm^2^).

The percentage ulcerated surface was calculated as the total area covered by all lesions expressed as a proportion of the total corpus mucosal surface area. The gastric mucus of each stomach was collected and weighed. The percentage of inhibition (% I) was calculated using the following formula:

$$ \%\mathrm{I}=\left(\mathrm{U}\mathrm{S}\mathrm{c}\hbox{-} \mathrm{U}\mathrm{S}\mathrm{t}\right)\times 100/\mathrm{U}\mathrm{S}\mathrm{c} $$

Where USc = ulcer surface area of control and USt = ulcer surface area of test animal.

### Indomethacin-induced ulcers

In order to ascertain whether the antiulcer properties of the aqueous and methanol extracts were mediated by the stimulation of cyclooxygenase activity, the indomethacin-induced model was utilized as described by [9]. Fifty-four animals fasted for 24 h received indomethacin (5 mg/kg. p.o) for five days. The animals were then divided into nine groups (n = 6) and treated once daily for another five days with the respective test solutions as given below:

- Group 1(negative control): 5mg/kg indomethacin + distilled water
- Group 2: 5mg/kg indomethacin + 50mg/kg Maalox
- Group 3: 5mg/kg indomethacin +100μg/kg Misoprostol
- Groups 4, 5, 6: 5mg/kg indomethacin + 125, 250 or 500mg/kg of aqueous extract respectively
- Groups 7, 8, 9: 5mg/kg indomethacin + 125, 250 or 500mg/kg of methanol extract respectively

After the test solutions administration, the rats were sacrificed on the sixth day, the stomachs were removed and opened along the greater curvature and washed. Gastric lesions were observed and the ulcer index was determined [10] as follows: ***1****(ulcerated area: 1-6 mm*^*2*^*),****2****(ulcerated area: 7-12 mm*^*2*^*),****3****(ulcerated area: 13-18 mm*^*2*^*),***4***(ulcerated area: 19-24 mm*^*2*^*),****5****(ulcerated area > 24 mm*^*2*^*).* The gastric mucus of each stomach was collected and weighed.

### Acetic acid-induced gastric ulcers

The acetic acid-induced model was utilized as described by [11]. Sixty male wistar rats fasted for 24 h were used in this experiment. Under anesthesia resulting from a Diazepam (5 mg/kg)/Ketamine (50 mg/kg) (2/1 v/v) mixture, a laparotomy was done on fifty four animals through a midline epigastric incision and the six animals remained were used as normal control group. After exposing the stomach, 0.05 ml (v/v) of 30 % acetic acid solution was injected into the subserosal layer in the glandular part of the anterior wall. The stomach was bathed with saline in order to avoid adherence to the external surface of the ulcerated region and the abdomen was then closed. One day after administration of acid, daily treatment began and animals were treated orally once a day for 14 consecutive days. Brifly, the animals were treated with the respective test solutions as given below:

- Group 1 represented the normal control group, which received 1 mL/100 g body weight distilled water
- Group 2 represented the negative control group, which received 1 mL/100 g body weight distilled water
- Groups 3 and 4 represent the positive control groups which received 1 mL/100 g body weight Maalox (50mg/kg) and Ranitidine (50mg/kg) solutions, respectively.
- Groups 5 to 7 received 1mL/100 g body weight aqueous extract at doses of 125, 250 and 500mg/kg respectively.
- Groups 8 to 10 received 1 mL/100 g body weight methanol extract at doses of 125, 250 and 500mg/kg. On the day 15, all groups were sacrificed. The blood was collected and the stomachs was removed. The blood was used to determine the nitric oxide level and the stomachs for evaluating the ulcerated area, mucus weight, nitric oxide level and histological assessement.

### Measurement of mucus production

Gastric mucus production was measured in rats subjected to HCl/ethanol, pylorus ligation and acetic acid according to the method described by Tan *et al.* [8]. Gastric mucosa of each rat was gently scraped using a glass slide, and the mucus obtained was carefully weighed using a sensitive digital electronic balance. This operation was performed by the same experimenter each time.

### Blood collection

The heparinized blood samples of the rats were obtained from abdominal artere (catheterization).

Immediately, the blood was submitted to centrifugation (3000 rpm) for 15 min. After the centrifugation, the plasma obtained was preserved at -18 °C until use for NO levels.

### Histological assessment

One part of the stomachs were preserved in 10 % formalin solution and followed by tissue dehydrated with alcohol and xylene. Then, each sample was embedded in paraffin wax, sectioned at 5 μm in slides prior for staining. Haematoxylin and eosin stain was used. The slides were examined under light microscope and recorded with 40× lenses.

### Nitric oxide dosage

After acetic acid induction, the heparinized blood and the homogenized stomach of the rats were used to measure the nitric oxide level in accordance with the method described by [12]. NO content was quantified by measuring nitrite/nitrate concentration using Griess assay and sodium nitrite was used as standard. In brief, gastric homogenates were centrifuged at 3900 rpm for 25 min. The supernatant obtained and plasma were each followed by the rapid addition of Griess reagent and the absorbance at 540 nm was measured. The results were expressed as μmol/g tissue.

### Statistical analysis

Results were expressed as mean ± standard deviation (s.d). For statistical analysis of data, multiple comparisons were carried out using one-way analysis of variance (ANOVA) followed by a Dunnett’s test for post-hoc analysis. Statistical significance was acceptable at a level of p < 0.05. Data analysis was achieved using the software progam GraphPad InStat.

## Results

### HCl/ethanol-induced gastric mucosal lesions

**Fig. 1:**
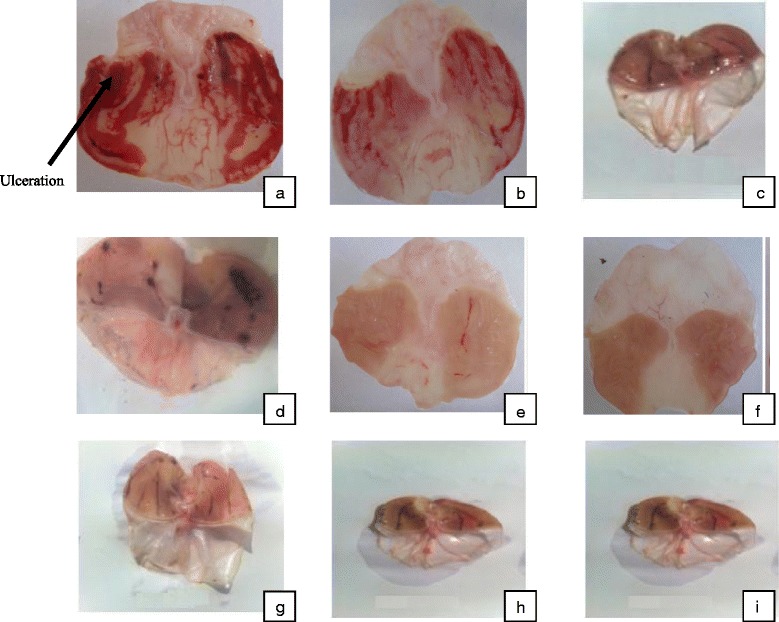
Gross appearance of the gastric mucosa in rats. **a** Rats pre-treated with 1ml/100g distilled water (ulcer control). Severe injuries are seen in the gastric mucosa: Hcl/ethanol produced extensive visible hemorrhagic necrosis of gastric mucosa. **b** & **c** Rats pre-treated with Maalox and Ranitidine (50 mg/kg) respectively: injuries to the gastric mucosa are milder compared to the injuries seen in the ulcer control rats. **d** & **e** Rats pre-treated with aqueous extract at doses 125 and 250 mg/kg respectively: moderate injuries are seen in the gastric mucosa, and the injuries decrease when the dose increase. **f** Rats pre-treated with aqueous extract at dose 500 mg/kg: no injuries are seen, so at this dose, the aqueous extract completely inhibits the gastric lesions induced by acidified ethanol. **g**, **h** & **i** Rats pre-treated with methanol extract at doses 125, 250 and 500 mg/kg: the injuries reduce with the increase of dose; hence, at 500 mg/kg few injuries are seen. The methanol extract reduces gastric lesions induced by acidified ethanol

The oral administration of HCl/ethanol solution produced characteristic lesions in the glandular portion of the negative control rat stomach with a total surface area of 236.54 ± 1.73 mm^2^. The aqueous extract of *Piptadeniastrum africanum* produced a dose-dependent inhibition of gastric ulceration ranging from 81.38 % at a dose of 125 mg/kg to 100 % at a dose of 500 mg/kg with ulcer surface areas of 58.86 ± 5.33 and 0.00 ± 0.00 mm^2^ respectively. The methanol extract also produced 75.83 % inhibition at a dose of 125 mg/kg and 96.52 % inhibition at a dose of 500 mg/kg. The corresponding ulcer surface areas were 85.74 ± 5.08 and 8.23 ± 2.47mm^2^. Animals treated with Maalox and Ranitidine at a dose 50mg/kg produced a significant (*p* < 0.01) decrease in ulcer surface area from 236.54 ± 1.73 mm^2^ to 67.74 ± 4.41 and 66.66 ± 9.78 mm^2^ respectively (Table 1). Macroscopic observation (Fig. 1) showed the gastric mucosal of rats.

**Table 1:** Effects of the stem bark aqueous and methanol extracts of Piptadeniastrum africanum on HCl/ethanol-induced gastric lesions in rats

Treatment	Dose (mg/kg)	n	US (mm^2^)	US %	UI	I %	Mucus weight (mg)
Distilled water	/	6	236.54 ± 1.73	14.04 ± 0.89	6.72 ± 0.32	/	152.27 ± 2.67
Maalox	50	6	67.74 ± 4.41**	4.33 ± 0.37**	4.27 ± 0.12**	80.91	182.25 ± 3.51
Ranitidine	50	6	66.66 ± 9.78**	4.27 ± 0.51**	5.23 ± 0.32**	81.15	130.25 ± 17.10
Aqueous extract of *P. africanum*	125	6	58.86 ± 5.33**	3.13 ± 0.34**	4.22 ± 0.23**	81.38	127.40 ± 5.20
	250	6	2.95 ± 0.76**	0.19 ± 0.05**	2.17 ± 0.17**	98.75	79.17 ± 5.23**
	500	6	0.00 ± 0.00**	0.00 ± 0.00**	0.00 ± 0.00pt>**	100.00	181.67 ± 19.22
Methanol extract of *P. africanum*	125	6	85.74 ± 5.18**	5.16 ± 0.29**	4.59 ± 0.18**	75.83	177.50 ± 4.74
	250	6	36.32 ± 5.96**	1.84 ± 0.22**	2.98 ± 0.34**	89.76	157.60 ± 5.42
	500	6	8.23 ± 2.40**	0.46 ± 0.13**	2.24 ± 0.30**	96.52	151.67 ± 4.20

n = number of animals per group, US = Ulcer surface, US % = Ulcer surface percentages, UI = Ulcer index, I % = inhibition percentage

**Significant difference at 0,01 in relation to the control group having received distilled water

### Indomethacin-induced ulcers

**Fig. 2:**
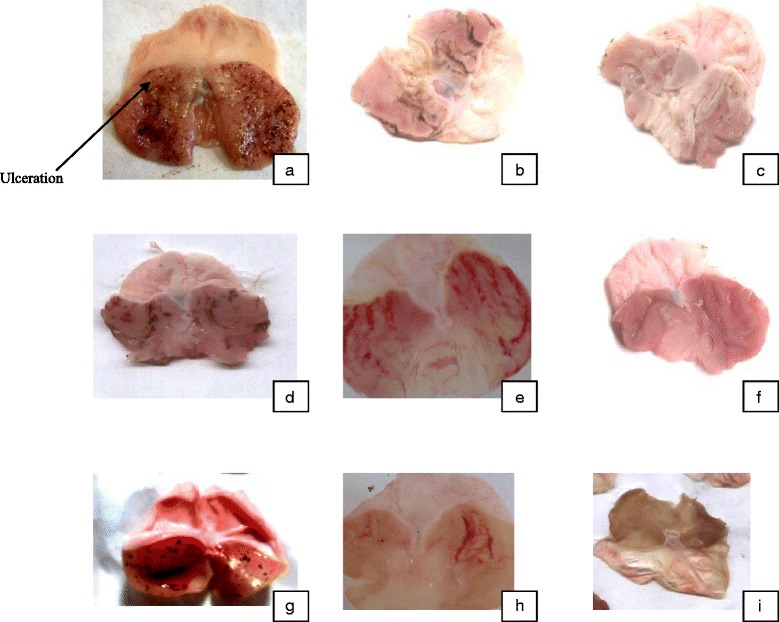
Macroscopic appearance of the gastric mucosa of the rats. **a** Rats pre-treated with 1ml/100g distilled water (ulcer control): severe injuries are seen in the gastric mucosa; Indomethacin produced extensive hemorrhagic necrosis of gastric mucosa. **b** & **c** Rats pre-treated with Maalox (50 mg/kg) and Misoprostol (100μg/kg) respectively: injuries to the gastric mucosa are milder compared to the injuries seen in the ulcer control rats. **d**, **e** & **f** Rats pre-treated with aqueous extract at doses 125, 250 and 500 mg/kg respectively: moderate injuries are seen in the gastric mucosa, and the injuries decrease when the dose increases; the aqueous extract reduces gastric lesions induced by indomethacin. **g**, **h** & **i** Rats pre-treated with methanol extract at doses 125, 250 and 500 mg/kg: the injuries reduce with the increase of dose; hence, at 500 mg/kg, few injuries are seen. The methanol extract reduces gastric lesions induced by indomethacin

Table 2 summarizes the results obtained in the experimental model of indomethacin-induced gastric ulceration in rats. The total surface area of ulceration obtained with control was 147.63 ± 5.58 mm^2^. The aqueous extract of stem bark of *Piptadeniastrum africanum* induced a significant (*p* < 0.01) decrease and dose-dependent of ulcer surface area from 147.63 ± 5.58 mm^2^ to 94.88 ± 0.93; 70.37 ± 1.97 and 2.09 ± 2.09 mm^2^ at doses of 125, 250 and 500 mg/kg, leading to inhibition percentage of 35.75; 52.23 and 98.58 % respectively. The methanol extract also reduced ulcer surface from 147.63 ± 5.58 mm^2^ to 97.86 ± 1.48; 70.90 ± 3.03 and 50.29 ± 2.75 mm^2^ at respective doses of 125, 250 and 500 mg/kg, corresponding to inhibition percentage of 33.71; 51.97 and 65.93 % respectively. Maalox (50 mg/kg) and Misoprostol (100 μg/kg) also reduced the gastric lesions induced by indomethacin, leading to the respective ulcer surfaces 71.80 ± 3.24 and 43.81 ± 3.07 mm^2^. The mucus weight of the control animals (45.33 ± 1.45 mg) was not significantly different (*p* > 0.05) from the mucus weight of the treated animals (Table 2). Gross appearance showed the gastric mucosa of the rats (Fig. 2).

**Table 2:** Effects of the stem bark aqueous and methanol extracts of Piptadeniastrum africanum on gastric lesions induced by indomethacin

Treatment	Dose (mg/kg)	n	US (mm^2^)	US %	UI	I %	Mucus weight (mg)
Distilled water	/	6	147.63 ± 5.58	10.05 ± 0.44	3.90 ± 0.29	/	45.33 ± 1.45
Maalox	50	6	71.80 ± 3.24**	4.82 ± 0.29**	1.94 ± 0.13^**^	51.36	47.50 ± 5.53
Misoprostol	0.10	6	43.81 ± 3.07**	3.18 ± 0.28**	1.57 ± 0.07**	70.36	45.67 ± 4.14
Aqueous extract of *P. africanum*	125	6	94.88 ± 0.93**	6.66 ± 0.20**	1.99 ± 0.15**	35.75	45.50 ± 1.98
	250	6	70.37 ± 1.97**	4.49 ± 0.24**	2.11 ± 0.18**	52.23	63.00 ± 7.52
	500	6	2.09 ± 2.09**	0.12 ± 0.12**	0.17 ± 0.17**	98.58	100.33 ± 10.08**
Methanol extract of *P. africanum*	125	6	97.86 ± 1.48**	6.21 ± 0.16**	2.53 ± 0.12**	33.71	53.50 ± 0.76
	250	6	70.90 ± 3.03**	3.97 ± 0.15**	1.63 ± 0.05**	51.97	69.67 ± 2.19**
	500	6	50.29 ± 2.75**	3.34 ± 0.21**	1.35 ± 0.05**	65.93	57.33 ± 1.31

n = number of animals per group, US = Ulcer surface, US % = Ulcer surface percentages, UI = Ulcer index, I % = inhibition percentage

**Significant difference at 0,01 in relation to the control group having received distilled water

### Acetic acid- induced gastric ulcers

**Fig. 3:**
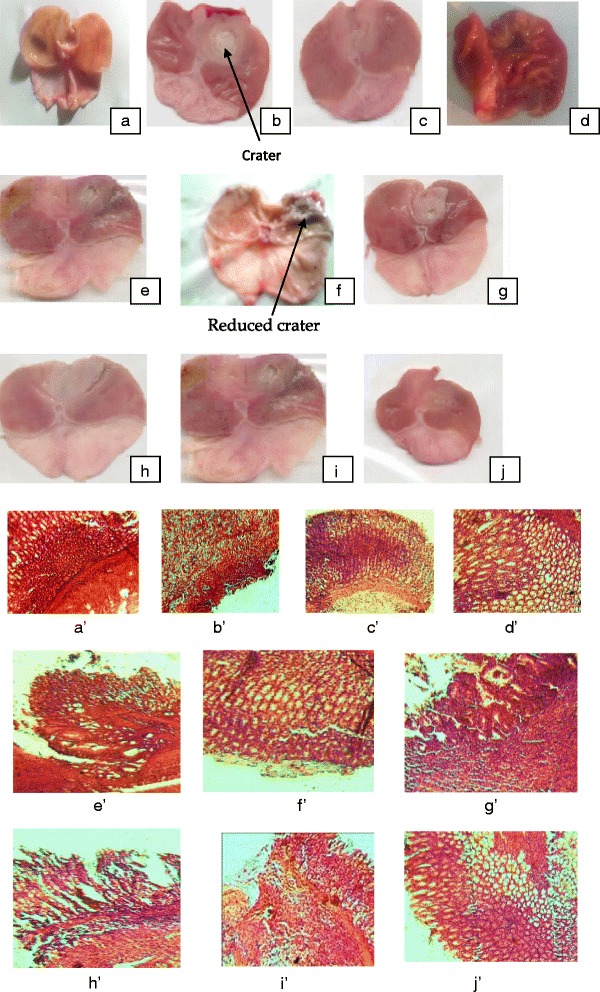
Macroscopic and histological study of acetic acid-induced gastric damage in rats. **a**’ & **a**: stomach and histological section of a normal control rat: no injuries to the gastric mucosa are seen and the gastric wall is normal. **b** & **b**’: stomach and histological section of a ulcer control rat: there is severe destruction of the surface epithelium and necrotic lesions penetrating deeply into mucosa and sub mucosa layer. **c** & **c**’: stomach and histological section of rat treated with Maalox (50 mg/kg): the gastric wall appears normally, but there is edema of mucosa and sub mucosa layer. **d** & **d**’: stomach and histological section of rat treated ranitidine (50 mg/kg): the gastric wall appears normally with all layers. **e** & **e**’: stomach and histological section of rat treated with 125 mg/kg of aqueous extract: there is mild disruption to the sub mucosal layer. **f**’ & **f**: stomach and histological section of rat treated with 250 mg/kg aqueous extract: there is moderate disruption to the surface epithelium. **g** & **g**’: stomach and histological section of rat treated with 500 mg/kg of aqueous extract: there is mild disruption to sub mucosal layer and edema of the muscle. **h** & **h**’: stomach and histological section of rat treated with 125 mg/kg of methanol extract: there is mild disruption to the epithelium surface and the sub mucosal layer and edema of the serosal layer. **i** & **i**’: stomach and histological section of rat treated with 250 mg/kg of methanol extract: there is mild disruption to the epithelium surface and edema of submucosal layer and serosal layer. **j** & **j**’: stomach and histological section of rat treated with 500 mg/kg of methanol extract: there is moderate disruption to the epithelium surface although the gastric wall appears normally

The administration of acetic acid to the gastric mucosa of rats is capable of producing a well-defined lesion. The healing effect of aqueous and methanol extracts of *Piptadeniastrum africanum* was demonstrated for the first time when the healing of chronic gastric ulcer induced by acetic acid in rats was accelerated. The total surface area of ulceration obtained with controls was 94.07 ± 7.99 mm^2^ (Fig. 3b). The oral administration of aqueous and methanol extracts at the doses of 125, 250 and 500 mg/kg for 14 consecutive days accelerated the healing of gastric ulcers in rats with a significant (*p* < 0.01) decrease in ulcer surface area from 94.07 ± 7.99 mm^2^ to 37.95 ± 1.06; 15.04 ± 0.84 and 38.21 ± 0.97mm^2^ for the aqueous extract and 28.00 ± 1.57; 42.15 ± 1.10 and 40.16 ± 0.65 mm^2^ for the methanol extract, respectively. Maalox and Ranitidine also reduced ulcer surface area significantly (*p* < 0.01) when compared with the control group. The mucus weight of control (177.17 ± 7.67 mg) significantly decreased when the aqueous and methanol extracts were administrated at the doses of 125, 250 and 500 mg/kg (Table 3).

**Table 3:** Effects of stem bark aqueous and methanol extracts of P. africanum on gastric ulcer induced by acetic acid

Treatment	Dose (mg/kg)	n	US (mm^2^)	Mucus weight (mg)	I %
Normal	/	6	0.00 ± 0.00**	107.00 ± 1.71*	/
Distilled water	/	6	94.07 ± 7.99	177.17 ± 7.67	/
Maalox	50	6	43.17 ± 1.05**	114.17 ± 1.14**	54.41
Ranitidine	50	6	12.56 ± 0.00**	109.00 ± 2.00**	86.73
Aqueous extract of *P. africanum*	125	6	37.95 ± 1.06**	130.17 ± 2.33**	59.92
	250	6	15.04 ± 0.84**	126.00 ± 1.48**	84.12
	500	6	38.21 ± 0.97**	134.50 ± 1.75**	59.65
Methanol extract *P. africanum*	125	6	28.00 ± 1.57**	151.17 ± 3.23**	70.43
	250	6	42.15 ± 1.10**	147.67 ± 9.63**	55.49
	500	6	40.16 ± 0.65**	139.33 ± 3.60**	57.59

n = number of animals per group, US = ulcer surface, I % = inhibition percentage

**Significant difference at 0,01 in relation to the control group having received distilled water

### Histological assessment

Histological examination of the stomachs removed from animals that were not treated with Maalox, Ranitidine or *P. africanum* showed complete ulceration (Fig. 3). However, a curative effect against ulceration was noticed in animals treated with Maalox, Ranitidine. Histopathology of stomach showed that animals that have received 250 mg/kg aqueous extract, 125, 250 and 500 mg/kg methanol extract of *P. africanum* reduced gastric lesion formation and sub mucosal edema similar to the Maalox and Ranitidine treated animals. Photomicrographs revealed that the mucosa of ulcer control animals have hemorrhagic erosion, discontinuity in the lining of epithelium cells and significant damage in sub mucosa. Normal mucosa with small strophic gland, mild hyperplasia and no edema were observed for animals treated with Maalox and Ranitidine. Similarly, mucosa of animals treated with 250 mg/kg aqueous extract, 125, 250 and 500 mg/kg methanol extract of *P. africanum* were normal with mild hyperplasia.

### Nitric oxide test

The results of this assay are listed in a Table 4. It appears that the latter animals treated aqueous extract at doses 250 and 500 mg/kg, the methanol extract at dose 125 mg/kg and the reference substances at a dose 50 mg/kg showed no significant difference (*p* > 0.05) in the concentration of plasma as compared to the negative control group that received distilled water. Except at the 125mg/kg dose of the aqueous extract, where there is a significant decrease (*p* < 0.05) and at doses 250 and 500 mg/kg where the methanol extract was rather a significant increase (*p* < 0.01) of this parameter.

**Table 4:** Effects of aqueous and methanol extracts of P. africanum on nitric oxide dosage

Treatment	Dose (mg/kg)	n	[NO] Plasma (μmol/ml)	[NO] Gastric supernatant (μmol/g)
Normal	/	6	3.09 ± 0.24	9.18 ± 0.71*
Distilled water	/	6	2.60 ± 0.22	12.96 ± 0.26
Maalox	50	6	3.40 ± 0.34	13.68 ± 0.67
Ranitidine	50	6	3.21 ± 0.33	7.66 ± 0.40**
Aqueous extract of *P. africanum*	125	6	1.64 ± 0.22*	16.91 ± 1.59**
	250	6	2.10 ± 0.22	13.78 ± 0.65
	500	6	2.71 ± 0.10	16.03 ± 0.76
Methanol extract of *P. africanum*	125	6	2.51 ± 0.17	15.23 ± 0.74
	250	6	7.30 ± 0.51**	11.17 ± 0.42
	500	6	4.65 ± 0.50**	13.88 ± 0.95

n = number of animals per group, [NO] = Nitric oxide concentration, *Significant difference at 0,05 in relation to the control group having received distilled water, **Significant difference at 0,01 in relation to the control group having received distilled water

## Discussion

Gastric ulcer result of an imbalance between aggressive and defensive factors of the gastric mucosa [1] factors. To consolidate this balance, different therapeutic agents including medicinal plants are used to reduce gastric acid secretion or enhance mucosal defense mechanisms through increased mucus production [13]. This study was conducted to evaluate the preventive and curative properties of aqueous and methanol extracts of stem bark of Piptadeniastrum *africanum* on gastric ulcer induced by HCl/ethanol mixture, indomethacin and acetic acid. The results obtained from this work showed that aqueous and methanol extracts at doses of 125, 250 and 500 mg/kg significantly reduced the ulceration of the surfaces against ulceration induced by these three models.

Necrotic mixture HCl/ethanol used to induce gastric ulcer is a quick and convenient method to highlight the cytoprotective properties of the extracts [14]. Indeed, ethanol drops the transmucosal potential difference and thus weakens the lining [15]. HCl in turn, accelerates the ulcerogenic process while enhancing lesions and reducing mucosal protection. Thus, aqueous and methanol extracts of stem bark of *Piptadeniastrum africanum* significantly inhibited (*p* < 0.01) and dose-dependently induced gastric lesions in rats, to the percentages of inhibition of 81.38; 98.75 and 100 % for the aqueous extract and then 75.83, 89.76 and 96.52 % for the methanol extract, respectively, at doses of 125, 250 and 500 mg/kg. In view of these results, the aqueous and methanol extracts showed significant cytoprotection. Moreover, [16] found similar results with aqueous and methanol extracts of *Gracinia indica* Linn. This could be due to a reduction of acid secretion or acid neutralization in the extracts [17] exceeding. In addition, the phytochemical screening of extracts shows the presence of alkaloids that are involved in the reduction of acidity [18].

Indomethacin is an anti-inflammatory drug known to induce gastric ulcers by inhibiting cyclo oxygenase which is characteristic of prostaglandin biosynthesis involved in maintaining the integrity of the gastric mucosa [19]. Prostaglandins promote the secretion of mucus and play a role of protection of the gastric mucosa. Thus, aqueous and methanol extracts of bark of *Piptadeniastrum africanum* reduce significantly (*p* < 0.01) and dose-dependently induced gastric lesions in rats, with percentage of cure (as it is a curative model) 35.75; 52.33 and 98.58 % for the aqueous extract, and 33.7; 51.97; and 65.93 to the methanol extract at the respective doses of 125, 250 and 500 mg/kg. Therefore, aqueous and methanol extracts of bark of *Piptadeniastrum africanum* have a cytoprotective effect against the gastric lesions induced by indomethacin. Since *Piptadeniastrum africanum* has been tested for its anti-inflammatory properties, the cytoprotective effect observed could be due to its anti-inflammatory activity [20]. Indeed, the damage of the gastric mucosa is also related to the increase in neutrophil infiltration into ulcerated tissues. These neutrophils inhibit ulcer healing mediated lipid peroxidation through the release of cytotoxic factors such as superoxide and hydrogen peroxide. Thus the removal of the infiltration of neutrophils by anti-inflammatory activity of the extracts could highlight a recovery mechanism [21].

Damage caused by acetic acid reached not only the mucous membrane and submucosa but also to the muscles; characteristic of chronic ulcer. The present study shows that the surface ulceration in animals treated with the extracts decreased significantly (*p* < 0.01) compared to that of control animals which received distilled water, corresponding to percentages of healing 59. 92; 84.12 and 59.65 % for the aqueous extract; and 70.43; 55.49 and 57.59 % for the methanol extract at the respective doses of 125, 250 and 500 mg/kg. In view of these percentages healing, aqueous and methanol extracts of *Piptadeniastrum africanum* accelerates the healing of chronic gastric ulcer. This could be due to the action of other factors such as growth factors. These growth factors have been identified as the first to have a healing effect on chronic gastric ulcer. They stimulate important cellular mechanisms such as migration and proliferation of epithelial cells that may have a cytoprotective effect by stimulating the release of prostaglandins. This is still true through histological sections of the stomach of rats which received aqueous and methanol extracts. Indeed, [22] working in cats showed that there was increased mucosal blood flow around the newly ulcerated region with a significant production of prostaglandins in this location area; this allows moving large quantities of glucose and oxygen necessary to the reconstitution of the cells. Thus the wall of the treated animals to Ranitidine showed complete reconstitution of tissues, as well as that treated at 250 mg/kg of the aqueous extract and then 250 and 500 mg/kg of the methanol extract. It is therefore possible that the aqueous and methanol extracts of *Piptadeniastrum africanum* act as Ranitidine and possess curative and healing effects.

Nitric oxide (NO) is a substance that plays an important role in maintaining the integrity of the mucosa as the synthesis of mucus and bicarbonate is dependent on it. The significant increase (*p* < 0.01) of the NO concentration observed in the plasma of animals treated with 250 and 500 mg/kg of the methanol extract, and then a significant reduction (*p* < 0.05) of those treated with 125 mg/kg of the aqueous extract showed that these extracts may well be the course of action mediated by NO.

## Conclusion

The ulcer preventive and protective activity demonstrated in the present study provides a strong support for the traditional use of this plant in the treatment of gastric ulcer. Further studies are required to confirm the exact mechanism underlining the ulcer healing and protecting property of the extracts and identify the chemical constituents responsible for it.
